# The safety and efficacy of botulinum toxin A on the treatment of depression

**DOI:** 10.1002/brb3.2333

**Published:** 2021-08-22

**Authors:** Qilin Zhang, Wenqi Wu, Yuxin Fan, Yang Li, Jing Liu, Yingying Xu, Caixia Jiang, Zhen Tang, Cong Cao, Tong Liu, Li‐Hua Chen, Hua Hu, Weifeng Luo

**Affiliations:** ^1^ Department of Neurology and Suzhou Clinical Research Center of Neurological Disease the Second Affiliated Hospital of Soochow University Suzhou China; ^2^ Department of Psychiatry, Suzhou Guangji Hospital the Affiliated Guangji Hospital of Soochow University Suzhou China; ^3^ Institute of Neuroscience Soochow University Suzhou China; ^4^ Jiangsu Key Laboratory of Translational Research and Therapy for Neuro‐Psychiatric‐Diseases Soochow University Suzhou China; ^5^ Institute of Pain Medicine and Special Environmental Medicine Nantong University Jiangsu China; ^6^ Department of Nutrition and Food Hygiene School of Public Health Nantong University Jiangsu China

**Keywords:** botulinum toxin A (BoNT/A), depression, efficacy, safety, sertraline

## Abstract

**Objectives:**

Effective strategy for the treatment of depression is limited. This study was to evaluate the safety and efficacy of botulinum toxin A (BoNT/A) in the treatment of depression.

**Methods:**

Seventy‐six patients were assigned to the BoNT/A group (*n* = 52) and sertraline control group (*n* = 24). For the BoNT/A group, BoNT/A was injected into the frowning muscle, depressor muscle, occipital frontalis muscle, lateral canthus, and bilateral temporal region at 20 sites. Five units per site and a total of 100 units of BoNT/A were given. Patients in the sertraline control group were medicated with sertraline 50–200 mg (114.58 ± 52.08 mg) per day. Depression was assessed by the 17‐item Hamilton Depression Scale (HAMD‐17), 14‐item Hamilton Anxiety Scale (HAMA‐14), Self‐rating Depression Scale (SDS), and Self‐rating Anxiety Scale (SAS). All participants were followed up for 12 weeks.

**Results:**

Scores of HAMD, HAMA, SDS, and SAS decreased significantly in both BoNT/A and sertraline groups after treatment for 12 weeks. Overall, there were no differences in decreased magnitude between the two groups (*p* > .05). The HAMA, SDS, and SAS results showed that the onset time of BoNT/A was earlier than that of sertraline. Side effects rates were 15.38% for BoNT/A and 33.33% for sertraline.

**Conclusion:**

This study demonstrated significant antidepressant effects of BoNT/A. The efficacy of BoNT/A was comparable with established antidepressant sertraline. The onset time of BoNT/A was earlier than sertraline, and the proportion of side effects was less than sertraline. Therefore, BoNT/A could be a safe and effective option for the treatment of depression.

## Graphical abstract

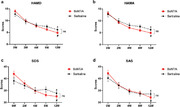

## 1 INTRODUCTION

Depression is a major cause of disability worldwide and immensely contributes to the global burden of diseases. According to the World Health Organization, over 300 million people have depression (Tomitaka et al., 2019). It is caused by complex interactions between psychological, social, and biological factors (Schotte et al., 2006). Depression is characterized by sadness, loss of appetite, hopelessness, and anxious feelings. These traits lead to a high rate of crippling, recrudescence, and suicide (Jun et al., 2014). However, due to the nonspecific symptomatology and variability in the severity of the disease, depression is reported to be underdiagnosed and undertreated (Starnawska et al., 2019). Patients are in a depressive state with low mood and an aversion to activity that affects a person's thoughts, behavior, feelings, and sense of well‐being, though patients in the depressive state may have not reached the diagnostic criteria for depressive disorder (Zou et al., 2017). Thus, an expert consensus on the diagnosis and treatment of anxiety, depression, and somatization symptoms in general hospitals in China defined criteria for the diagnosis of “depressive state” (Neuropsychology and Behavioral Neurology Group, Neurology Branch of Chinese Medical Association, 2016). It is acknowledged that the clinical manifestations of a depressive state may vary. However, the most common symptoms may include low mood, anhedonia, sleep disorders, cognitive impairment, sexual dysfunction, and gastrointestinal diseases (Zhao et al., 2020).

Currently, the underlying etiology and pathophysiology of depression remain largely unknown. Various hypotheses about depression pathogenesis have been proposed and tested. The most popular hypothesis of depression pathogenesis is called the “monoamine hypothesis,” which suggests that depression results from an imbalance of neurotransmitters such as norepinephrine, serotonin, and dopamine (Wu et al., 2015). The “monoamine hypothesis” appears to be supported by the mechanism of action of antidepressants. Thus, the most common available treatment for patients with depressive state is medicating with antidepressants affecting monoaminergic transmission accordingly. However, exhaustive explorations have failed to find a specific dysfunctional monoamine system for individual depressed people (Boku et al., 2018). Thus, over one‐third of patients have a low response to monoamine‐based antidepressant treatments (Chan et al., 2020). Moreover, these antidepressants are limited by their slow and poor effects (Huang et al., 2017). Therefore, it is urgent and of great significance to find a novel method to deal with depression.

Another hypothesis called the “emotion hypothesis” was proposed by Charles Darwin and William James (Adelmann & Zajonc, 1989; Ludwig & Welch, 2019), and was further advanced as the “emotional proprioception hypothesis” by Finzi and Rosenthal (2016). These hypotheses assume facial expression muscles are associated with emotion‐related areas in the brain and provide a theoretical basis for our current study. Botulinum toxin A (BoNT/A) is a natural neurotoxin that could paralyze facial muscle fibers and be used for aesthetic enhancement (Heckmann et al., 2003). Thus, several relevant studies have examined the efficacy of BoNT/A in the treatment of depression. However, those studies have limitations such as patients with varying severity of depression, small sample size, lack of proper control group, and so on (Chugh et al., 2018; Hexsel et al., 2013; Magid et al., 2014; Wollmer et al., 2012). Therefore, based on previous studies, this study enrolled patients with lighter diseases that met the diagnosis of “depressive state” according to the expert consensus mentioned above, increased the sample size, and set up established antidepressant sertraline as a positive control group to assess the safety and efficacy of BoNT/A for treatment of depression.

## 2 MATERIALS AND METHODS

### 2.1 Study population

Patients diagnosed with “depressive state” in the Department of Neurology of the Second Affiliated Hospital of Soochow University from February 2017 to September 2019 were recruited. Because few patients met the diagnostic criteria for “depression,” we chose participants with mild to moderate symptoms, who met meet the diagnostic criteria “depressive state.” This study was registered with the Chinese Clinical Trial Registry (ChiCTR1800019802).

Inclusion criteria as follows: (1) All patients who met the diagnosis of “depressive state” in the expert consensus (Neuropsychology and Behavioral Neurology Group, Neurology Branch of Chinese Medical Association, 2016). (A) Typical symptoms: low mood, loss of interest and pleasure, lack of energy, or fatigue; (B) Common symptoms: decreased attention, decreased self‐evaluation, self‐guilt, and feelings of worthlessness, pessimism, self‐injury/suicide thoughts or behaviors, sleep disorders, and loss of appetite; (C) the course of the disease was more than 2 weeks. (2) The score of the 17‐item Hamilton Depression Scale (HAMD‐17) (Hamilton, 1960): 7 ≤ HAMD‐17 ≤ 24.

Exclusion criteria as follows: (1) Patients who suffered from severe mental diseases other than depressive state; (2) patients who suffered from depression caused by other systemic diseases or brain diseases; (3) patients having the previous history of brain injuries, seizures, or other nervous system diseases; (4) patients having the previous history of serious systemic diseases such as the cardiovascular system, respiratory system, and immune system; (5) patients having the previous history of alcohol or morphine abuse; (6) patients having previous hepatic or renal dysfunction; (7) pregnant or lactating women.

This study adopted a prospective, nonrandomized controlled design. All protocols were approved by the Medical Ethics Committee of the Second Affiliated Hospital of Suzhou University (number: JD‑LK‑2017‑011‑02). All patients signed informed consent.

### 2.2 Pharmacological intervention

**FIGURE 1:**
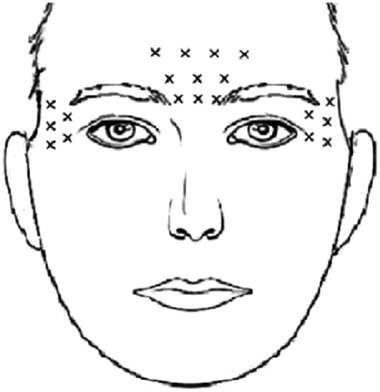
Schematic representation of BoNT/A injection sites. (X stands for the injection site, 10 points for frowning muscle, depressor muscle, and occipital frontalis muscle, five points each for lateral canthus, and bilateral temporal region. A total of 20 sites and five units per site were applied for the BoNT/A.)

For the BoNT/A injection group: patients were treated with freeze‐dried crystal BoNT/A (trade name: Hengli, Cat. No. S10970037, origin: Lanzhou, China). A total of 100 units BoNT/A were diluted into 0.9% saline, and a final volume of 2 mL was obtained. Based on previous studies (Magid et al., 2014), a total of 10 sites were injected at one time into frowning muscle, depressor muscle, occipital frontalis muscle. In addition, five sites each at the lateral canthus of the eyes and bilateral temporal region were chosen to inject BoNT/A. A total of 20 sites and five units per site were applied for the BoNT/A (Figure 1).

For the sertraline positive control group: sertraline is a selective serotonin reuptake inhibitor (SSRI) and is one of the first‐line agents for treating depression (Kato et al., 2018). Patients were treated with sertraline hydrochloride (trade name: Zoloft, Cat. No.H10480191, origin: The United States) 50–200 mg (114.58 ± 52.08 mg) once a day in the morning for 12 weeks. Depression was assessed using HAMD‐17, the 14‐item Hamilton Anxiety Scale (HAMA‐14), the Self‐Rating Depression Scale (SDS) (Zung, 1965), and the Self‐Rating Anxiety Scale (SAS) (Zung, 1971). All participants were followed up for 12 weeks, and depression‐related scales were assessed at baseline and 2, 4, 8, 12 weeks after therapy.

All data were processed with the SPSS 17.0 statistical software. *p*‐Values    < .05 were considered significant. The scores of HAMD, HAMA, SDS, and SAS before and after treatment with BoNT/A or sertraline were compared by two‐way repeated‐measured ANOVA following the Bonferroni post hoc correction. The differences between the two groups were determined using an unpaired *t*‐test. One‐way ANOVA following by the Bonferroni post hoc correction was used to compare scores at different follow‐up time points. The results were presented by mean ± SEM.

## 3 RESULTS

### 3.1 Baseline characteristics

The baseline characteristics of participants were presented in Table 1. After the assessment of HAMD scores, a total of 76 patients were recruited for the trial. Fifty‐two patients were assigned to the BoNT/A treatment group and 24 to the sertraline control group. The BoNT/A and sertraline groups did not differ significantly in terms of age, gender, HAMA, HAMD, SDS, and SAS at baseline. All participants completed the trial up to the final visit.

**TABLE 1:** Baseline characteristics of participants

	BoNT/A (*n* = 52)	Sertraline (*n* = 24)	*p*	Unpaired *t*‐test
Age (Year)	56.04 ± 10.95	53.83 ± 11.54	.425	0.802
Gender (female, %)	43 (82.69%)	18 (75%)	.537^a^	
HAMD	14.04 ± 5.16	12.79 ± 4.40	.310	1.023
HAMA	13.17 ± 5.55	12.75 ± 5.27	.755	0.314
SDS	41.98 ± 8.92	39.08 ± 6.51	.159	1.424
SAS	42.29 ± 9.23	40.25 ± 7.16	.342	0.956

Abbreviations: BoNT/A, botulinum toxin A; HAMA, Hamilton Depression Scale; HAMD, Hamilton Depression Scale; SAS, Self‐Rating Anxiety Scale; SDS, Self‐Rating Depression Scale.

^a^Fisher's exact test.

### 3.2 Efficiency of BoNT/A

**FIGURE 2:**
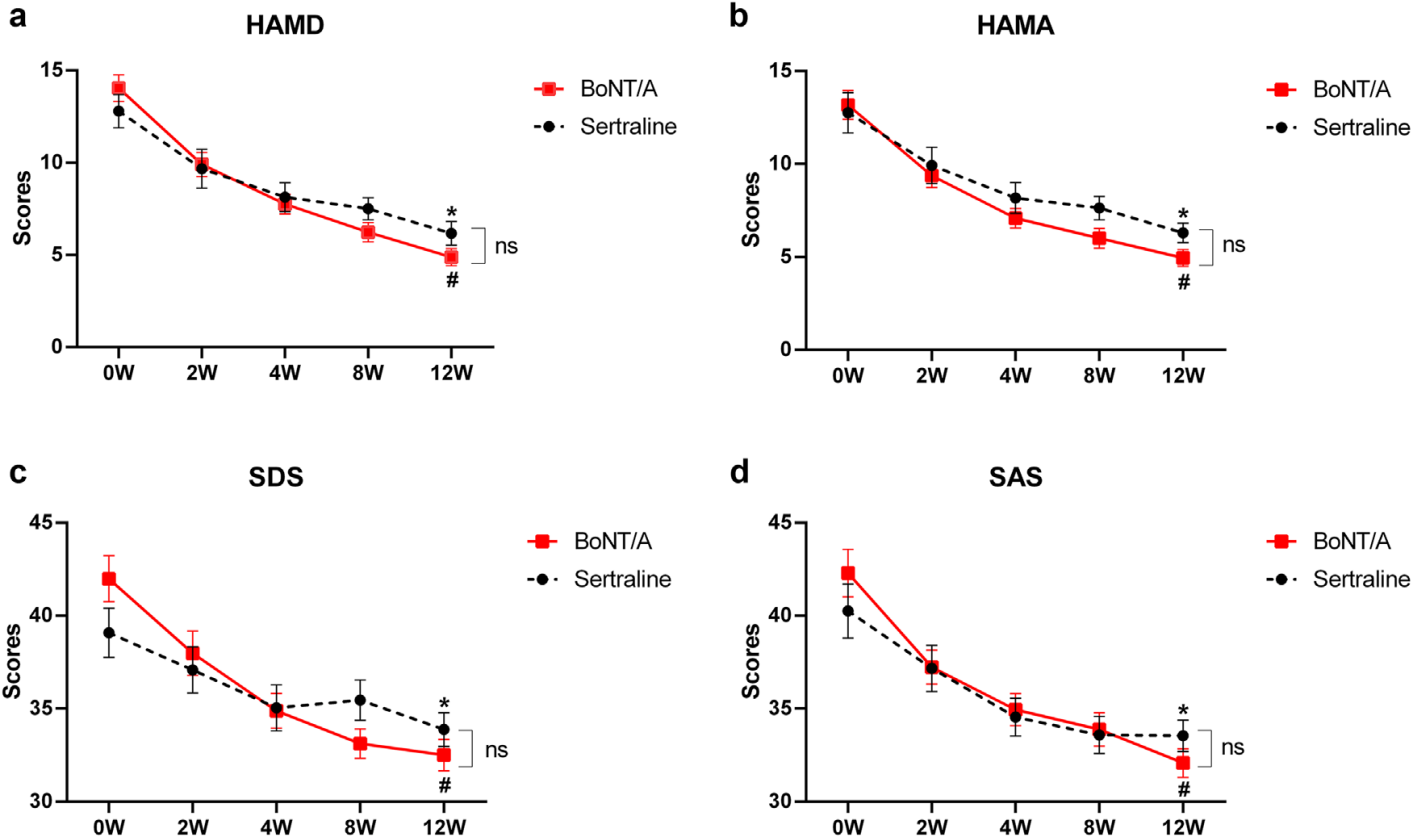
BoNT/A and sertraline decreased the scores of HAMD, HAMA, SDS, and SAS after 12 weeks of therapy when comparing with baseline (a, HAMD; b, HAMA; c, SDS; d, SAS). The scores of HAMD, HAMA, SDS, and SAS of BoNT/A and sertraline groups were determined using two‐way repeated‐measured ANOVA (#, *: there was statistical significance between scores of 12 weeks and baseline, *p* < .05, # for the BoNT/A group, * for the sertraline group). The difference between BoNT/A and sertraline groups at each time point was determined using the unpaired *t*‐test (ns: there were no statistical significance between BoNT/A and sertraline). BoNT/A, botulinum toxin A; HAMD, Hamilton Depression Scale; HAMA, Hamilton Depression Scale, SDS, Self‐Rating Depression Scale; SAS, Self‐Rating Anxiety Scale

During 12 weeks of follow‐up, the scores of HAMD, HAMA, SDS, and SAS in both BoNT/A and sertraline groups decreased significantly after treatment (*p* < .0001). After intervention for 12 weeks using BoNT/A and sertraline, the HAMD score decreased to 4.89 ± 3.33 and 6.17 ± 3.13, respectively. These scores were below the diagnosis score of “depressive state,” which implied that the participants of both groups had recovered after treatment. It is important to note that there was no difference in the therapeutic effect between BoNT/A and sertraline treatment overall (*p* > .05) (Figure 2).

**FIGURE 3:**
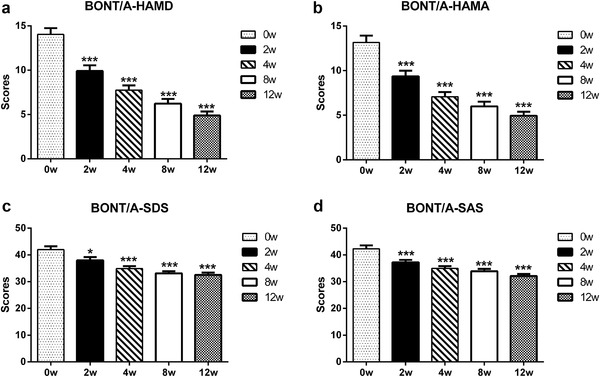
Comparison of HAMD, HAMA, SDS, and SAS scores in the BoNT/A group after therapy with the baseline. (a, HAMD; b, HAMA; c, SDS; d, SAS). The scores of HAMD, HAMA, SDS, and SAS were compared by one‐way ANOVA. BoNT/A, botulinum toxin A; HAMD, Hamilton Depression Scale; HAMA, Hamilton Depression Scale; SDS, Self‐Rating Depression Scale; SAS, Self‐Rating Anxiety Scale. * *p* < .05; *** *p* < .0001

**FIGURE 4:**
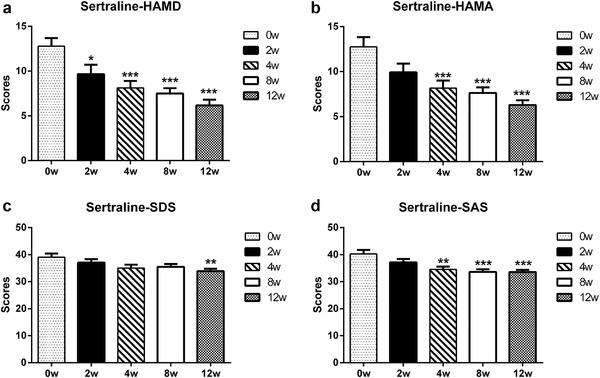
Comparison of HAMD, HAMA, SDS, and SAS scores in the sertraline group after therapy with the baseline. (a, HAMD; b, HAMA; c, SDS; d, SAS). The scores of HAMD, HAMA, SDS, and SAS were compared by one‐way ANOVA. HAMD, Hamilton Depression Scale; HAMA, Hamilton Depression Scale; SDS, Self‐rating Depression Scale; SAS, Self‐rating Anxiety Scale. * *p* < .05; ** *p* < .01; *** *p* < .0001

The HAMD scores showed onset time of both BoNT/A and sertraline groups was the second week. Compared with the scores of baselines, the decline magnitude in the BoNT/A treatment group (decreased magnitude: 29.48%) was greater than in the sertraline control group (decreased magnitude: 24.42%). The onset time of BoNT/A was earlier than sertraline. As shown in Figures 3 and 4, the onset time for BoNT/A was revealed in the second week, while the onset time for sertraline was revealed in the fourth week for HAMA. For SDS, the onset time for BoNT/A was the second week, while the onset time for sertraline was the 12th week. For SAS, the onset time for BoNT/A was the second week, while the onset time for sertraline was the fourth week.

### 3.3 Side effects

**FIGURE 5:**
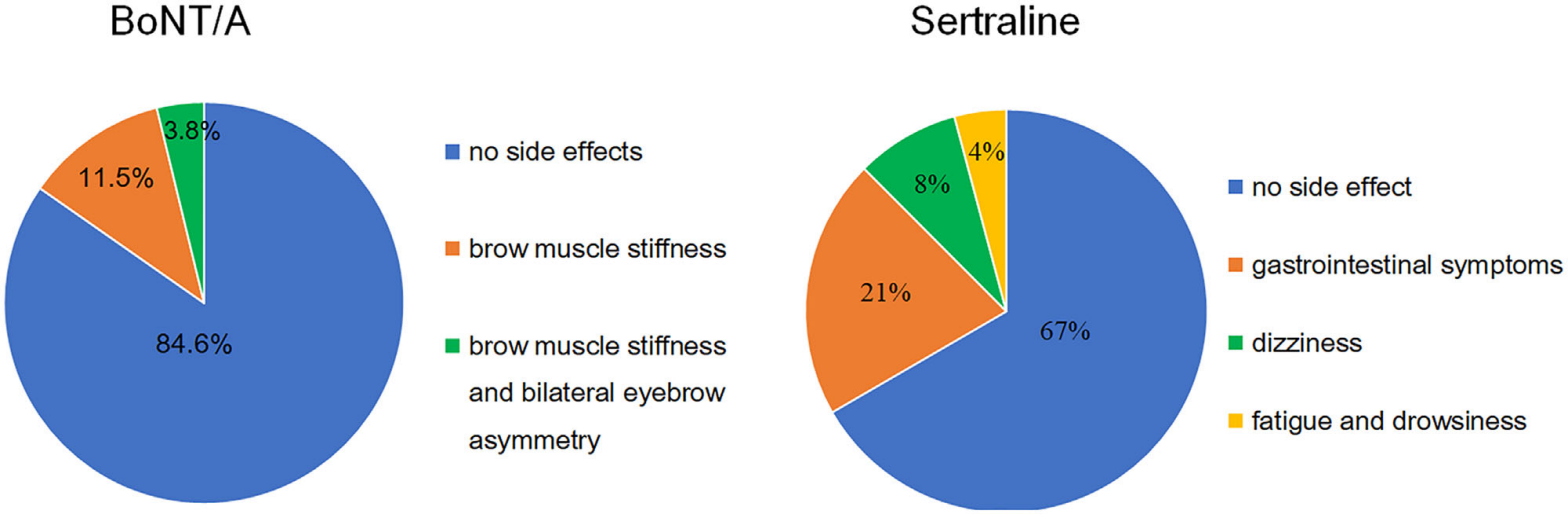
Proportions of side effects occurred in the BoNT/A and sertraline treatment group. BoNT/A: botulinum toxin A

Of the 52 patients who received BoNT/A, eight (15.4%) showed brow muscles stiffness. Two of the eight patients with brow muscles stiffness also had bilateral eyebrow asymmetry. Seven patients completely recovered within 4 weeks and one patient recovered within 6 weeks. Of the 24 patients who received sertraline, eight (33%) showed side effects (Figure 5). Gastrointestinal symptoms such as nausea and dyspepsia occurred in five cases (21%), dizziness in two cases (8%), and fatigue and drowsiness in one case (4%). These symptoms completely disappeared after 2 weeks of drug tolerance.

## 4 DISCUSSION

The finding of this study showed that BoNT/A significantly decreased the scores of mood‐related scales during 12 weeks of treatment and demonstrated the antidepressant effects of BoNT/A. The efficacy of BoNT/A was comparable with the established antidepressant drug sertraline. Here, it was further revealed that the onset time of BoNT/A was earlier than sertraline and the proportion of side effects was less than sertraline.

Our study is based on the hypothesis of the “facial feedback” theory, which supports facial expressions can affect emotional perceptions. The hypothesis further states that emotional facial expression is associated with an emotional state, such as happy or sad states (Nakatani & Yamaguchi, 2014). Facial expression muscles are also important for encoding and transmitting information to the cerebral emotional loop. The afferent nerve fibers of peripheral muscle can transmit emotion‐related information to the brain and inform the brain of the current emotional state (Heller et al., 2014; Kim et al., 2014; Lee et al., 2012). The BoNT/A can block the cholinergic synapse between the lateral branches of motor neurons and Renshaw cells (Marchand‐Pauvert et al., 2013) and inhibit the release of acetylcholine at the neuromuscular junction. This leads to temporary denervation of the affected muscle, which finally leads to typical flaccid paralysis in the injected muscle (Heckmann et al., 2003). Therefore, injecting BoNT/A into the muscles associated with negative emotions like sadness and fear may significantly improve patients' depressive symptoms. Using functional magnetic resonance imaging, Hennenlotter et al. found the activity of the left amygdala decreased when patients mimicked angry facial expressions after receiving BoNT/A in the frowning muscle (Hennenlotter et al., 2009).

In this study, on administering BoNT/A, the facial expression muscle fibers were paralyzed and significant relief from depressive symptoms was revealed among the patients. These findings are in line with previous studies. For example, Finzi and Rosenthal found the depressive symptoms of patients were significantly improved by injecting BoNT/A into the frowning muscle among 10 patients recruited (Finzi & Rosenthal, 2014). Chugh et et al. recruited 42 patients with depressive disorder and confirmed the antidepressant effect of injecting BoNT/A into the frowning muscle (Chugh et al., 2018). Hexsel et al. aimed to evaluate symptoms of self‐esteem and depression before and after BoNT/A injections in the glabella in depressed and nondepressed patients. They observed that the depressed patients showed significant improvement in depression symptoms after BoNT/A injections. They suggested that BoNT/A was a potential treatment to improve depression symptoms in patients with major depressive disorder (Hexsel et al., 2013).

Oral monoamine antidepressants are commonly used in the clinical treatment of depressive disorders. BoNT/A, as a possible new therapy for depression, needs to be compared with established antidepressants. Based on the fact that monoamine antidepressants act on cholinergic neurons similar to BoNT/A (Li et al., 2018), our study used sertraline as the positive control group. Sertraline, an SSRI, is widely recognized and used because it is safe and efficient (Keller et al., 1998; Keppel Hesselink et al., 1992). Results of the therapeutic effects of sertraline and BoNT/A revealed that the antidepressant effect of BoNT/A was equivalent to sertraline, and the effect of treatment of BoNT/A was better as indicated by the shorter onset time.

The selection of injection points for BoNT/A also played an important role in its curative efficacy. For nearly a century, researchers confirmed frowning muscle, depressor muscle, and frontal muscle were related to negative emotions, while musculus and temporal orbicularis oculi muscles were related to positive emotions (Adelmann & Zajonc, 1989). Another study found that botulinum toxin injected into creases may reduce negative facial expressions by restricting frowning and other negative expressions. However, the toxin had no effects on positive emotions (Alam et al., 2008). Most of the injection points in the previous studies were 5 points focusing on frowning muscle and depressor muscle (Finzi & Rosenthal, 2014; Finzi & Wasserman, 2006; Hexsel et al., 2013; Magid et al., 2014; Wollmer et al., 2012). In this study, in addition to the frowning muscle and depressor muscle, the occipital frontalis muscle, lateral canthus, and bilateral temporal region were selected for injection. These sites were preferred because the denervation of facial muscles may reduce the sensory information transmitted from the trigeminal meridian bundle to the brainstem to reduce the coupling function between the brainstem and the left amygdala (Finzi & Rosenthal, 2016).

Although this study along with other's studies showed the effectiveness of BoNT/A on depression, the mechanisms underlying this therapeutic effect are still unknown. An experiment on mice found BoNT/A could significantly improve the depressive performance of mice performed the forcing swimming test, tail suspension test, and open field test (Li et al., 2019). The depression ameliorating effects of BoNT/A was associated with increased expression levels of 5‐hydroxytryptamine and brain‐derived neurotrophic factor (BDNF) in mice brain and BDNF/extracellular signal regulated kinase(ERK)/cyclic AMP response element (CRE)‐binding protein (CREB) pathways (Li et al., 2019). More research is needed to determine whether BoNT/A can regulate neurotransmitters in patients with depression.

At present, injecting BoNT/A into wrinkles between eyebrows and crow's feet has been confirmed to be safe practice (Carruthers et al., 2015). As a widely used cosmetic method, the safety of BoNT/A injected into the facial muscles around the eyes has been approved (Molina et al., 2015). The incidence of side effects after injection is low, and the most common side effects are headache (1.1%) and eye‐related events (0.4%), like blepharoptosis, eyelid edema, dry eye, and periocular muscle convulsions. However, the duration is short, and the symptoms are tolerable for patients (Molina et al., 2015). In this study, eight of 52 patients who received BoNT/A had the side effect of brow muscles stiffness (15.38%). Eight of 24 (33.33%) patients who received sertraline had side effects. The BoNT/A treatment group had less side effects with milder symptoms and shorter duration, which further supported the safety of BoNT/A as compared with the sertraline control group.

The efficacy and safety of BoNT/A as a treatment for depression were confirmed in this study, but there were still some limitations: first, although the number of patients recruited was more than previous studies, the sample size was still not big enough and most of the patients were female. However, the therapeutic effects of BoNT/A on male patients were good. This supported the previous observation that there was no sex difference of BoNT/A in treating depression (Chugh et al., 2018). Second, because patients with major depressive disorder have complex conditions and poor compliance, all the cases enrolled in our study were patients with mild to moderate symptoms diagnosed with a depressive state. Whether BoNT/A is effective in patients with severe depressive disorders to be explored. Third, this study used a positive control as opposed to a placebo control.

In summary, this study demonstrated significant antidepressant effects of BoNT/A. The efficacy of BoNT/A was comparable with established antidepressant sertraline. The onset time of BoNT/A was earlier than sertraline, and the proportion of side effects was less than sertraline. Therefore, BoNT/A can be a safe and effective option for treating patients with depressive state. Further mechanistic studies are needed to provide a molecular basis for the efficacy of BoNT/A in treating depression.

## CONFLICT OF INTEREST

The authors declare they have no relevant interests.

## AUTHORS CONTRIBUTION

Qilin Zhang: data curation, formal analysis, writing ‐ original draft; Wenqi Wu: data curation, methodology, writing ‐ original draft; Yuxin Fan: data curation, formal analysis, writing ‐ original draft; Yang Li: writing ‐ review and editing; Jing Liu: analysis and interpretation of data; Yingying Xu: data curation, formal analysis; Caixia Jiang: methodology; Zhen Tang: methodology; Cong Cao: statistical analysis, manuscript revision; Liu Tong: statistical analysis, manuscript revision; Li‐Hua Chen: statistical analysis, manuscript revision; Hua Hu: investigation, manuscript revision; Weifeng Luo: study design, supervision, manuscript revision.
